# Genome-Wide Profiling of *Diadegma semiclausum* Ichnovirus Integration in Parasitized *Plutella xylostella* Hemocytes Identifies Host Integration Motifs and Insertion Sites

**DOI:** 10.3389/fmicb.2020.608346

**Published:** 2021-01-15

**Authors:** Ze-hua Wang, Yue-nan Zhou, Jing Yang, Xi-qian Ye, Min Shi, Jian-hua Huang, Xue-xin Chen

**Affiliations:** ^1^Institute of Insect Sciences, College of Agriculture and Biotechnology, Zhejiang University, Hangzhou, China; ^2^Ministry of Agriculture Key Laboratory of Molecular Biology of Crop Pathogens and Insects, Zhejiang University, Hangzhou, China; ^3^Zhejiang Provincial Key Lab of Biology of Crop Pathogens and Insects, Zhejiang University, Hangzhou, China; ^4^State Key Laboratory of Rice Biology, Zhejiang University, Hangzhou, China

**Keywords:** host integration motif, *Plutella xylostella*, *Diadegma semiclausum* ichnovirus, integration, Polydnavirus

## Abstract

Polydnaviruses (PDVs), classified into two genera, bracoviruses (BVs) and ichnoviruses (IVs), are large, double-stranded DNA viruses, which are beneficial symbionts of parasitoid wasps. PDVs do not replicate in their infected lepidopteran hosts. BV circles have been demonstrated to be integrated into host genomic DNA after natural parasitization. However, the integrations of IV circles *in vivo* remain largely unknown. Here, we analyzed the integration of *Diadegma semiclausum* ichnovirus (DsIV) in the genomic DNA of parasitized *Plutella xylostella* hemocytes. We found that DsIV circles are present in host hemocytes with non-integrated and integrated forms. Moreover, DsIV integrates its DNA circles into the host genome by two distinct strategies, conservatively, and randomly. We also found that four conserved-broken circles share similar motifs containing two reverse complementary repeats at their breaking sites, which were host integration motifs (HIMs). We also predicted HIMs of eight circles from other ichnoviruses, indicating that a HIM-mediated specific mechanism was conserved in IV integrations. Investigation of DsIV circle insertion sites of the host genome revealed the enrichment of microhomologies between the host genome and the DsIV circles at integration breakpoints. These findings will deepen our understanding of the infections of PDVs, especially IVs.

## Introduction

Endoparasitoid wasps, one of the most species-rich animal groups on Earth, have developed various strategies to regulate their host’s physiology and development to ensure successful parasitism (Pennacchio and Strand, 2006; Ye et al., 2018). Polydnaviruses (PDVs) are obligatory symbionts of parasitoid wasps and are essential for the successful parasitism of thousands of species of parasitoid wasps. PDV particles with multiple segments of double-stranded, superhelical DNAs were first observed about 50 years ago (Rotheram, 1967; Vinson and Scott, 1975). PDVs were classified into two genera, bracoviruses (BVs) and ichnoviruses (IVs), respectively, associated with the two largest parasitoid groups, Braconidae and Ichneumonidae (Francki et al., 1991). PDVs are present in the wasp genome as integrated proviruses (Webb, 1998; Strand and Burke, 2014). The assembly and replication of PDV virions that are injected into lepidopteran hosts during parasitoid oviposition occur only in the nuclei of ovarian calyx cells of female wasps (Gruber et al., 1996). PDVs infect most of the host immune cells and many other tissue cells as well after parasitization (Strand, 1994; Beck et al., 2007; Bitra et al., 2011), but they do not propagate themselves in the host cells.

So far, 13 PDVs, including eight BVs (Wyder et al., 2002; Espagne et al., 2004; Choi et al., 2005; Webb et al., 2006; Desjardins et al., 2008; Chen et al., 2011; Jancek et al., 2013; Yu et al., 2016) and five IVs (Webb et al., 2006; Lapointe et al., 2007; Tanaka et al., 2007; Djoumad et al., 2013; Doremus et al., 2014), have been fully sequenced since the report of the first PDV, *C. congregata* bracovirus (CcBV) (Espagne et al., 2004). The encapsidated genomes of PDVs do not contain genes coding for replication and particle production, thereby impeding particle replication in caterpillar hosts during parasitism. However, PDV circles are reported to integrate into genomic DNA of host hemocytes after natural parasitization (Chevignon et al., 2018). Virulence genes are transcribed in the infected host cells, resulting in the expression of virulence proteins that can suppress immune responses and disrupt the development of the parasitized caterpillar hosts thereby ensuring wasp development (Theilmann and Summers, 1986; Strand and Burke, 2013, 2014; Ye et al., 2018). PDV-produced miRNA is also reported to arrest host growth by modulating expression of the host ecdysone receptor (Wang et al., 2018).

Polydnaviruses do not replicate within their lepidopteran hosts, but viral genes are expressed throughout parasitism, which raised the questions of whether and how they persist in the hosts. Some of these PDV circles were suggested to persist as episomes (Strand et al., 1992; Webb and Strand, 2005) in the caterpillar host, but recent studies also revealed the presence of chromosomally integrated forms in host-derived cultured cells (Gundersen-Rindal and Dougherty, 2000; Volkoff et al., 2001; Gundersen-Rindal and Lynn, 2003; Doucet et al., 2007; Beck et al., 2011). Studies performed on BVs, especially *Glyptapanteles indiensis* BV (GiBV), *Microplitis demolitor* BV (MdBV), and CcBV, identified a motif, named host integration motif (HIM), that mediates the insertion of viral circles into the genome of the lepidopteran hosts *Lymantria dispar*, *Pseudoplusia includens*, and *Manduca sexta*, respectively (Gundersen-Rindal and Lynn, 2003; Beck et al., 2011; Herniou et al., 2013; Chevignon et al., 2018). Two MdBV circles and eight CcBV circles have been formally demonstrated to be integrated into host genomic DNA after natural parasitization (Beck et al., 2011; Chevignon et al., 2018). As to the integrations of IVs, only *Tranosema rostrale* ichnovirus (TrIV) circle F was reported to integrate into the genomic DNA of the host *Choristoneura fumiferana* CF-124T cells *in vitro* (Doucet et al., 2007). However, the integrations of IV circles *in vivo* remain largely unknown.

In this study, we analyzed the integration of *Diadegma semiclausum* ichnovirus (DsIV), a PDV of the wasp *D. semiclausum* (Haliday) that is a larval parasitoid of the diamondback moth, *Plutella xylostella* (Linnaeus), one of the most important pests of cruciferous crops worldwide. We found that DsIV circles persist in parasitized *P. xylostella* hemocytes with two different forms, circular and integrated, through high-throughput sequencing analysis. The integrated DsIV circles integrate their DNA into the host genome by two distinct strategies, conservatively, and randomly. We identify four HIMs from DsIV and predict eight HIMs from other ichnoviruses and show that the HIMs of ichnoviruses have two pairs of boundary sequences forming reverse complementary repeats. We further found that the integrations of DsIV circles show a preference for the host genome regions that contained overlapping sequences of their HIMs.

## Materials and Methods

### Insect Rearing and Parasitization

*Plutella xylostella* and its endoparasitoid *D. semiclausum* were reared as previously described (Huang et al., 2008). They were maintained at 25 ± 1°C, with 65% relative humidity, and a 14-h light/10-h dark cycle. Adult wasps were fed with 20% honey/water (*V*/*V*). Late 3rd instar *P. xylostella* host larvae were individually exposed to a single *D. semiclausum* female within a 10 mm × 80 mm tube to ensure 100% parasitization.

### Genome Resequencing of Parasitized *P. xylostella* Hemocytes

Hemocytes of about 500 parasitized *P. xylostella* larvae at 24 h post parasitization (pp) were collected as one group. Genomic DNA from three independent replicates was isolated using the Puregene Core kit (Qiagen). DNA concentration was assessed by NanoDrop^®^ spectrophotometers (Thermo Fisher, MA, United States). A total amount of 1 μg genomic DNA per group was used as input for the library preparation. The sequencing libraries were generated using the VAHTS Universal DNA Library Prep Kit for Illumina^®^ (Vazyme, Nanjing, China) following the manufacturer’s recommendations and were sequenced on an Illumina HiSeq X Ten platform with 150 bp paired-end module.

### Read Mapping and Data Analysis

A total of 1.84 billion clean reads were obtained from the three Illumina runs (Supplementary Table 1). All clean reads were mapped against the DsIV genome (GenBank No. KF156214–KF156260) using BLASTN (*E*-value <10^–5^). A total of 1,256,800 reads were mapped to 47 DsIV circles (Supplementary Table 2). In a stringent analysis, only reads mapped with at least 20 nucleotides were kept to avoid incorrect mapping due to short alignments. DsIV-related reads were then mapped against the *P. xylostella* genome (You et al., 2013) to identify specifically chimeric reads. Again, only reads that mapped to the *P. xylostella* genome with a size above 20 nucleotides were kept. A qualified chimeric read must contain both DsIV and *P. xylostella* sequences, and the two sequences are on the opposite sides. The sum of the base numbers of their mapped sequences should be more than 140 bp. A total of 1,435 chimeric reads corresponding to DsIV circles were obtained based on the strict filtering criteria (Table 1). We also performed the mapping analysis by using Burrows–Wheeler alignment (BWA) tools, version 0.7.10, and only 797 chimeric reads were sorted out. While comparing the dataset with the results from BLASTN analysis, the 797 chimeric reads were all covered. Since the 1,435 chimeric reads were confirmed to have both the virus and the caterpillar genome sequences, we decided to use the dataset with the large amount to do the further analysis. Reads were then mapped again to the DsIV genome to identify junction sites of DsIV circles and to the *P. xylostella* genome to identify integration sites. For an example, if the 1–51 bases of a chimeric read were mapped to the circle of DsIV-15 (2048–1988 nt) and the 50–150 bases were mapped to the scaffold_206 of *P. xylostella* genome (80256–80156 nt), it means that DsIV-15 viral circle is linearized at the position of 1987–1988 nt and it integrates into host genome at the site of 80256–80257 nt within the scaffold_206. The sequences of DsIV and *P. xylostella* host within the same chimeric read are shown in Supplementary Data 1.

**TABLE 1:** The numbers of chimeric reads for different DsIV circles.

**DsIV ID**	**Related reads**	**DsIV ID**	**Related reads**
DsIV-01	0	DsIV-25	14
DsIV-02	8	DsIV-26	25
DsIV-03	3	DsIV-27	1
DsIV-04	12	DsIV-28	21
DsIV-05	10	DsIV-29	15
DsIV-06	17	DsIV-30	18
DsIV-07	36	DsIV-31	21
DsIV-08	5	DsIV-32	1
DsIV-09	17	DsIV-33	129
DsIV-10	12	DsIV-34	9
DsIV-11	9	DsIV-35	31
DsIV-12	5	DsIV-36	23
DsIV-13	1	DsIV-37	0
DsIV-14	7	DsIV-38	47
DsIV-15	541	DsIV-39	13
DsIV-16	3	DsIV-40	42
DsIV-17	33	DsIV-41	35
DsIV-18	14	DsIV-42	48
DsIV-19	24	DsIV-43	3
DsIV-20	0	DsIV-44	17
DsIV-21	69	DsIV-45	13
DsIV-22	6	DsIV-46	15
DsIV-23	17	DsIV-47	17
DsIV-24	28		

To test the robustness of the method and its ratio of false positives, we additionally analyzed the reads from a non-parasitized host with the same algorithm. We screened the chimeric reads among five datasets of the whole genome of non-parasitized *P. xylostella* with NCBI SRA numbers: ERR2508315, ERR2508316, ERR2508317, ERR2508318, and ERR2512126. The results showed only three reads were mapped to DsIV genome, which confirms that our data is accurate and the BLASTN method is suitable in this study.

### The Percent of Each Integrated DsIV Circle

We first estimated the depth for each DsIV circle according to the DsIV-related reads before calculating the percent of each integrated DsIV circle. The depth was calculated as follows: *x* = (the number of each DsIV-related reads × 150 bp) / the size of each DsIV circle. When one DsIV circle integrates into a host genome, two junction sites will be produced. So, the percent of each integrated DsIV circle is calculated as follows: % = (the number of chimeric reads / 2) / the depth of each circle × 100. The threshold used to differentiate circle forms was defined as 1%. For each integrated circle, the ratio of conservatively integrated form = the number of chimeric reads indicating the same junction site of each DsIV circle / the total chimeric reads of each DsIV circle.

### Verification of DsIV Host Integration Motifs

Two DsIV circles (DsIV-15 and DsIV-40) were chosen to confirm the locations of HIMs identified by chimeric reads according to PCR-based detection (Beck et al., 2011); DsIV-13 and DsIV-21 lacking HIMs were used as controls. Briefly, DsIV-13, 15, 21, and 40 were divided into 4, 4, 5, and 5 amplicons, respectively, by designing overlapping primer pairs (Supplementary Table 4) that specifically amplified regions of different DsIV circles (Figure 3A). Genomic DNA from hemocytes of parasitized *P. xylostella* (24 h pp) was used as a template, while genomic DNA isolated from female *D. semiclausum* ovaries was used as a control. PCRs were then run in 20-μl reaction mixtures containing 0.2 μM of each specific primer, 10 ng of template DNA, and 1 unit of LA Taq polymerase (TaKaRa, Tokyo, Japan). Cycling conditions were as follows: initial denaturation step at 94°C for 2 min, followed by 30 cycles of denaturation at 94°C for 20 s, annealing at 55°C for 20 s, and extension at 72°C for 2 min, with a final extension step at 72°C for 10 min. The amplified products were analyzed on 1.0% agarose gels.

### Identification of Candidate HIMs in Other Ichnovirus Circles

Host integration motif sequences of four DsIV circles (DsIV-15, 33, 38, and 40) were used to search for similar motifs among *Tranosema rostrale* IV (TrIV), *Hyposoter fugitivus* IV (HfIV), *Glypta fumiferanae* IV (GfIV), *Campoletis sonorensis* IV (CsIV), and *Apophua simplicipes* IV (AsIV) circles by BLASTN analyses (*E*-value < 10, identity >80%, and length >15). The database used for BLASTN analyses contained 41 segments of TrIV, 56 segments of HfIV, 106 segments of GfIV, 24 segments of CsIV, and 226 segments of AsIV, which were downloaded from NCBI and the accession numbers are listed in Supplementary Table 5. Alignment analyses of HIM regions were performed with MEGA 7.0 software, and the visualizations of the alignments were made using Jalview (Waterhouse et al., 2009).

### Data Availability

The raw data for genome resequencing of parasitized *P. xylostella* hemocytes have been deposited at the SRA database of NCBI with accession numbers SRR11880655, SRR11880656, and SRR11880657. The sequences of each chimeric read are shown in Supplementary Data 1.

## Results

### DsIV Circles Are Present in Two Different Forms in *P. xylostella* Hemocytes

**FIGURE 1:**
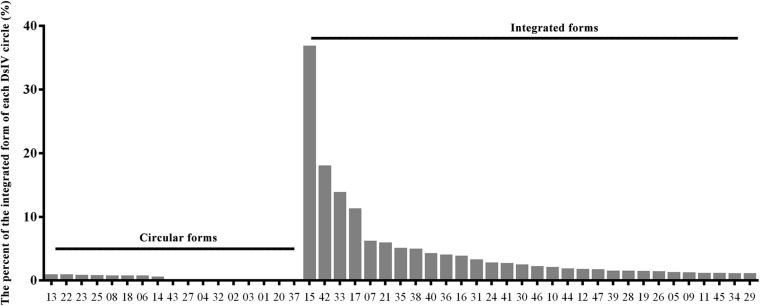
DsIV circles are present in different chromosomally integrated and circular forms in *P. xylostella* hemocytes at 24 h post parasitization (pp). The abscissa represents different circles of *Diadegma semiclausum* ichnovirus (DsIV), and the ordinate represents the percent of each integrated DsIV circle (%).

Genomic DNAs were isolated from hemocytes of parasitized *P. xylostella* larvae and were deep-sequenced to analyze the integration pattern of each DsIV circle. A total of 1.84 billion clean reads were obtained from three independent experiments (Supplementary Table 1). Among them, 1,256,800 reads were aligned to 47 DsIV circles (Supplementary Table 2). The number of DsIV-related reads mapping to each DsIV circle was variable (Supplementary Table 2). For example, 214,609 reads were aligned to DsIV-02, while only 877 reads were aligned to DsIV-16 (Supplementary Table 2). DsIV-related reads were then mapped against the *P. xylostella* genome to identify chimeric reads. A total of 1,435 chimeric reads containing both nucleotides of DsIV sequence and *P. xylostella* sequence were sorted out (Table 1 and Supplementary Data 1). We found that the numbers of chimeric reads mapping to different circles varied widely (Table 1). For example, the number of chimeric reads mapping to DsIV-15 was 541, while no chimeric reads mapped to DsIV-01, 20, or 37 (Table 1). The circles for which a higher number of chimeric reads was found did not correspond to circles that are more abundant within the particles (Supplementary Table 2). As the number of chimeric reads is related to the integration efficiency of the circles, we determined the integrations of DsIV circles by analyzing the percentage of each integrated DsIV circle. We found that DsIV circles are present in two different forms in *P. xylostella* hemocytes at 24 h pp. There were 17 “non-integrated” circles (Figure 1). On the contrary, the other 30 DsIV circles were present in both circular and integrated forms (Figure 1).

### Two Distinct Integration Strategies of DsIV Circles

**FIGURE 2:**
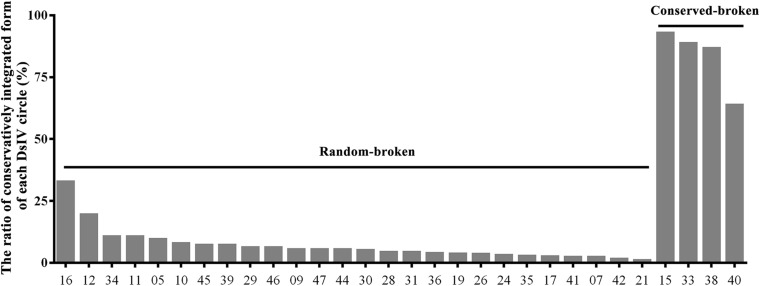
The ratio of the same fractural site when *Diadegma semiclausum* ichnovirus (DsIV) circles were integrated. The abscissa represents different circles of DsIV, and the ordinate represents the frequency of fracture (%), arranged in the order of the breaking frequency (small to large).

Based on the alignment of chimeric reads on the DsIV genome, we could easily figure out the junction sites for integrated DsIV circles (Supplementary Figure 1). The integrated circles can be divided into two categories according to the ratio of conservatively integrated forms of each DsIV circle. One type we referred as “conserved-broken circle” (ratio >50%), which means that the DsIV circles are linearized at a particular site of their sequence, and the other type we called “random-broken circle” (ratio <50%), which means that the DsIV circles are linearized randomly (Figure 2). Specifically, four DsIV circles (15, 33, 38, and 40) had the particular site of their circles, which were named as conserved-broken circles (CBCs) (Figure 2). The remaining 26 integrated circles were linearized completely randomly during integration, and therefore, they were named “random-broken circles” (RBCs) (Figure 2). CBCs can also integrate randomly into the host genome (Supplementary Table 3). The data combined with the results from alignments of chimeric reads and the DsIV genome show that CBC integration is associated with the deletion of a stretch of nts (32 to 311 bp) in each DsIV circle (Table 2).

**TABLE 2:** Sites where DsIV circles are linearized.

**DsIV ID**	**DsIV size (bp)**	**Accession numbers**	**Upstream DsIV nt position**	**Downstream DsIV nt position**	**Length (nt)**
DsIV-15	4,024	KF156228.1	1943–1944	1997–1998	54
DsIV-33	5,277	KF156246.1	1058–1059	1090–1091	32
DsIV-38	4,756	KF156251.1	2391–2392	2702–2703	311
DsIV-40	5,127	KF156253.1	373–374	449–450	76

### Validation of HIM Locations Using a PCR-Based Detection Assay

**FIGURE 3:**
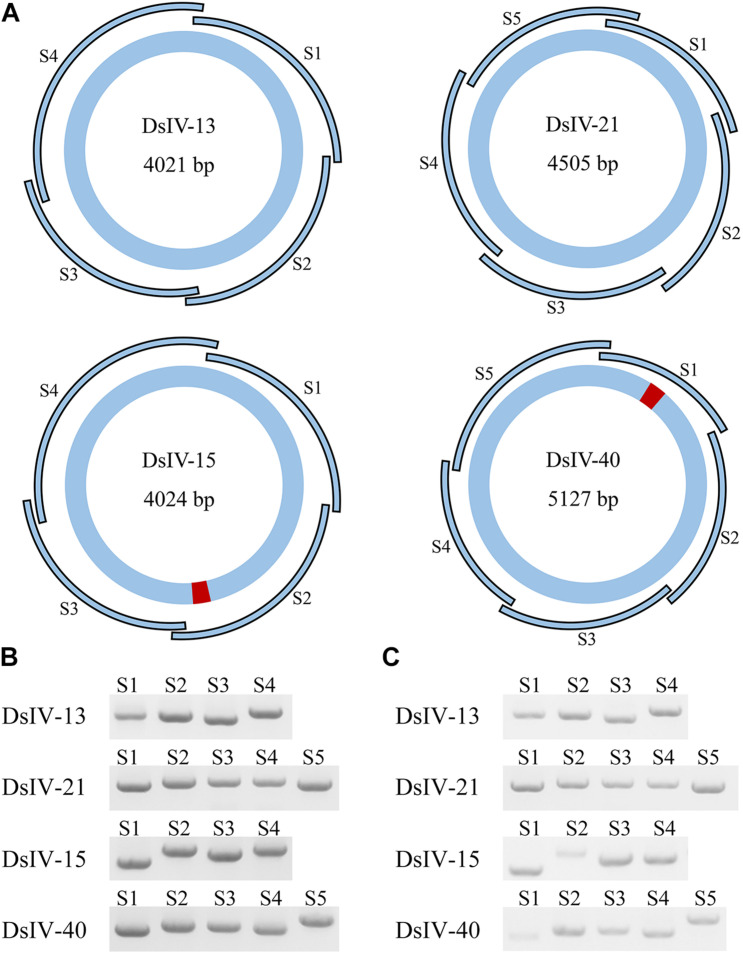
PCR-based detection assay to confirm the locations of host integration motif (HIM) regions. **(A)** The designs of PCR-based integration assays for *Diadegma semiclausum* ichnovirus (DsIV)-13, 15, 21, and 40. DsIV-13 and DsIV-21 do not have HIM regions, while DsIV-15 and DsIV-40 have HIMs, which are shown in red. The segment size is shown inside each DsIV circle. The PCR fragments of each circle are labeled as S1, S2, S3, and so forth. There are four fragments for DsIV-13, five fragments for DsIV-21, four fragments for DsIV-15, and five fragments for DsIV-40 in total. PCR products of different fragments of each circle with genomic DNA from female *D. semiclausum* ovaries **(B)** and hemocytes of parasitized *P. xylostella* at 24 h post parasitization (pp) **(C)** were used as templates.

Previous studies performed on BVs identified a motif, HIM, that mediated the insertion of viral circles into their host genomes. We selected two CBCs (DsIV-15 and DsIV-40) and used a PCR-based assay to validate the locations of their HIMs identified by chimeric reads. One “non-integrated” circle (DsIV-13) and one RBC (DsIV-21) were selected as negative controls (devoid of HIM). Primers were designed to amplify specific regions from each circle (Figure 3A). When using the genomic DNA of wasp ovaries as a template, PCR products were obtained for all pairs of primers (Figure 3B). In contrast, very few amplicons were obtained for DsIV-15-S2 and DsIV-40-S1 regions using templates isolated from host hemocytes 24 h pp (Figure 3C). The results suggest that DsIV-15 is disrupted into linear DNAs at its S2 region, and DsIV-40 is disrupted at its S1 region during integration, which was expected because DsIV-15 S2 and DsIV-40 S1 contain the HIM sequences.

### The Structure of HIMs From Ichnoviruses

**FIGURE 4:**
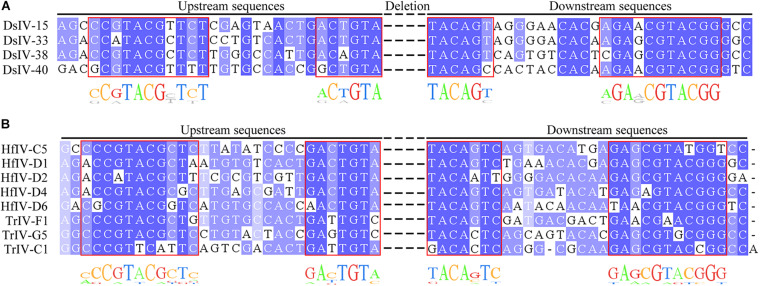
Alignment of host integration motifs (HIMs) of DsIV circles and other IV homologous sequences. Alignment of HIM sequences with similarity for each site colored in shades of blue for DsIV **(A)**, HfIV, and TrIV **(B)**.

We investigated the structure of DsIV HIMs from four CBCs. Alignment analysis showed that they had two pairs of boundary sequences forming reverse complementary repeats of 11 nts (CCGTACGCTCT and AGAGCGTACGG) and 6 nts (ACTGTA and TACAGT) constituting the borders of the insertions (Figure 4A). Further, the DsIV HIM sequences were used to identify the candidate HIMs in other IV circles from TrIV, HfIV, GfIV, CsIV, and AsIV. Finally, we identified candidate HIMs from five HfIV and three TrIV circles (Table 3). The distance between two putative junction sites of each circle ranges from 32 to 1,781 bp (Table 3), which is similar to what is observed in DsIV. Among circles containing predicted HIMs, TrIV-F1 was reported to be integrated into *Choristoneura fumiferana* CF-124T cells (Doucet et al., 2007), and the breaking site of TrIV-F1 is consistent with our results (Figure 4B). Alignment analysis of eight predicted HIMs showed that they also had two pairs of boundary sequences forming similar reverse complementary repeats (Figure 4B).

**TABLE 3:** Information of the predicted host integration motif of other IVs.

**Ichnoviruses**	**Wasp hosts**	**Circle ID**	**Accession numbers**	**Putative junction sites**		**Putative deletion**
				**Upstream nt position**	**Downstream nt position**	**(nt)**
HfIV	*H. fugitivus*	C5	AB291183.1	920–921	952–953	33
		D1	AB291196.1	854–855	886–887	33
		D2	AB291197.1	1768–1769	1800–1801	33
		D4	AB291199.1	4556–4557	4600–4601	45
		D6	AB291200.1	364–365	441–442	78
TrIV	*T. rostrale*	F1	AF421353.1	2194–2195	2226–2227	33
		G5	AB291163.1	5094–5095	6874–6875	1,781
		C1	AY940454.1	1456–1457	1487–1488	32

### The Integration Sites of CBCs

**FIGURE 5:**
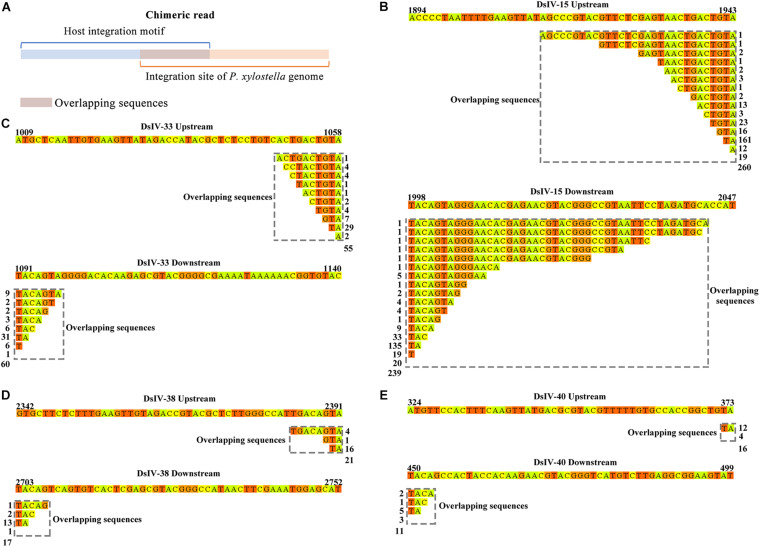
The integration sites of conserved-broken circles. **(A)** The overlapping sequence of the chimeric read shows the integration site of the host genome. The integration sites of DsIV-15 **(B)**, DsIV-33 **(C)**, DsIV-38 **(D)**, and DsIV-40 **(E)**. The upstream and downstream sequences of junction sites of DsIV circles are marked by colors and the overlapping sequences are marked by gray dotted boxes. The side numbers represent the read counts of each kind of chimeric read, which have the same overlapping sequences.

To determine whether DsIV circle integration occurred randomly or in preferential regions of the *P. xylostella* genome, we analyzed the integration sites of four CBCs, which can be obtained according to the chimeric reads. However, we did not observe any regions of the *P. xylostella* genome in which DsIV circle integration preferentially occurred. We anticipated whether there is a specific shared motif in the *P. xylostella* genome near the different insertion sites for any DsIV circles. We analyzed 499 chimeric reads of DsIV-15, 115 chimeric reads of DsIV-33, 38 chimeric reads of DsIV-38, and 27 chimeric reads of DsIV-40. As shown in Figure 5A, we found that there was generally an overlapping sequence in the middle of each chimeric read. We counted and classified the overlapping sequences of chimeric reads. The results showed that these four circles preferred to integrate into the host genome regions that contained overlapping sequences of their HIM regions (Figure 5).

## Discussion

Polydnaviruses are large, double-stranded DNA viruses that constitute a unique virus family, Polydnaviridae, and are classified into two genera: bracoviruses (BVs) and ichnoviruses (IVs) (Francki et al., 1991). PDVs influence two major areas of host biology: immunity and development (Kim et al., 2013; Ignesti et al., 2018; Wang et al., 2018; Ye et al., 2018), which are essential for the successful parasitism of parasitoid wasps. Though the functions of BVs and IVs injected into their hosts during parasitism are similar, there are many differences between them, including morphology and origins (Gundersen-Rindal et al., 2013; Strand and Burke, 2014; Ye et al., 2014).

The DNA circles in BV or IV virions injected into hosts do not contain genes coding for particle production, thereby impeding viral replication, which raised the question of how viral DNA persists in lepidopteran hosts. In addition, the question of persistence was also raised by the expression of the genes throughout parasitism. Some PDV circles were suggested to persist as episomes (Strand et al., 1992; Webb and Strand, 2005), and other pieces of evidence indicate that part of these PDV circles integrate into the genomes of host insect cells *in vitro* or *in vivo* (Gundersen-Rindal and Dougherty, 2000; Gundersen-Rindal and Lynn, 2003; Doucet et al., 2007; Beck et al., 2011; Chevignon et al., 2018). Eight out of nine tested circles of CcBV were integrated in *M. sexta* hemocyte genomic DNA (Beck et al., 2011; Chevignon et al., 2018), which indicates that some other circles of CcBV may be present in lepidopteran hosts as non-integrated forms. We analyzed the integration of 47 DsIV circles in parasitized *P. xylostella* hemocytes using high-throughput sequencing and found that DsIV circles persist in *P. xylostella* at 24 h pp in two forms, i.e., non-integrated and integrated forms.

It should be noted that there were huge variations in the numbers of aligned reads mapping to each DsIV circle. This situation indirectly reflects the various abundance of DsIV circles in wasp ovaries as reported for MdBV and CcBV (Beck et al., 2007; Chevignon et al., 2014). Though no relationship between circle abundance and existing forms in lepidopteran hosts was observed, we found that the top nine most abundant DsIV circles persisted in the non-integrated form at 24 h pp. We only detected the integrations of DsIV circles in the host hemocytes at 24 h pp, which neglects the integrations in other tissues or at other time points.

It was reported that integrations of BV circles involve a HIM (Beck et al., 2007; Chevignon et al., 2014). Interestingly, we found that 26 DsIV circles integrated into the *P. xylostella* genome by two distinct strategies, conservatively and randomly. However, the HIMs only mediated the integrations of four CBCs while the remaining 26 DsIV circles had no HIMs and were broken randomly during integration, which we named RBCs. In this study, the number of chimeric reads from 4 CBCs is 759 and the number of chimeric reads from 26 RBCs is 567, which suggest that the efficiency of HIM-mediated integrations of CBCs is higher than that of RBCs. In addition, we also get extra 109 chimeric reads for the remaining 17 DsIV circles. Due to the limitations of the few number of the reads, we cannot predict the integration models for those 17 circles. Alternatively, we did try Manta (Version1.6.0) for identifying the integration sites in host genome, but it failed probably because of the very low frequency of integration events of DsIV circles. Further studies may be needed to figure out why DsIV has such a low insertion frequency and what are the integration patterns for all DsIV circles. Till now, HIMs were identified in several BV species, including GiBV, MdBV, and CcBV (Gundersen-Rindal and Lynn, 2003; Beck et al., 2011; Herniou et al., 2013; Chevignon et al., 2018). However, as to the integrations of IVs, only one TrIV circle was reported as integrated into its host genome (Doucet et al., 2007), which makes it impossible to reveal the conserved structure of IV HIMs. We identified four HIMs of DsIV circles and thus found that the HIMs had two pairs of boundary sequences forming reverse complementary repeats constituting the borders of the insertions according to the alignment analysis. The reverse complementary repeats also exist in the HIMs from BVs (Beck et al., 2011; Chevignon et al., 2018).

However, the reverse complementary repeats of BV HIMs are different from those of IV HIMs (Supplementary Figure 2). In particular, both CcBV and MdBV HIMs share similar reverse complementary repeats consisting of palindromic sequences of 9 bp (GAAAATTTC and GAAATTTTC) and 5 bp (CTAGT and ACTAG) in MdBV and 8 bp (TAAATTTC and GAAATTTA) and 5 bp (CTGGT and ACCAG) in CcBV (Chevignon et al., 2018). However, the reverse complementary repeats in the HIMs of DsIV circles consist of 11 bp (CCGTACGCTCT and AGAGCGTACGG) and 6 bp (ACTGTA and TACAGT). During this study, we also identified eight candidate HIMs from HfIV and TrIV. Moreover, the HIM of TrIV-F1 mediates the integration that was reported (Doucet et al., 2007). It is unsurprising that similar HIMs are not found for GfIV and AsIV, whose associated wasps belong to Banchinae. However, it is inexplicable that HIMs are not found for CsIV, whose associated wasp belongs to Campopleginae. The genome resequencing of MdBV identified 10 additional circles (Burke et al., 2014). Thus, the resequencing of the CsIV genome may find circles containing similar HIMs. However, the biological relevance of the structures of HIMs is uncertain. It is hypothesized that the HIMs more probably correspond to similar protein-binding sites, resulting in the assembly of a nucleoprotein complex (Chevignon et al., 2018). Further work is required to uncover the mechanism of PDV integrations mediated by HIMs.

Data obtained on the integration of CcBV shows that the insertion events are widespread in *M. sexta* hemocyte DNA (Chevignon et al., 2018), which is consistent with results found in DsIV based on the analysis of chimeric reads. However, we reveal a specific shared motif in the *P. xylostella* genome near the different insertion sites for DsIV circles, which was not found in the integrations of CcBV (Chevignon et al., 2018). In our dataset, we observed a significant enrichment of microhomologies between the host genome and the DsIV circles at integration breakpoints, which is similar to what was observed in human papillomavirus integration (Hu et al., 2015).

In summary, our results demonstrated that the DsIV circles integrate into the host genome by two distinct strategies. HIMs were identified from IVs, which could mediate integration of these particular circles. Furthermore, the enrichment of microhomologies between the host genome and the DsIV circles at integration breakpoints was observed. These findings will deepen the understanding of how PDV circles persist in the hosts.

## Summary

Parasitoid wasps are a species-rich group of animals that live in or on other arthropods. Polydnaviruses (PDVs), divided into two genera, bracoviruses, and ichnoviruses, are double-stranded DNA viruses associated with parasitic wasps (primary hosts), which do not replicate in their infected caterpillar hosts (secondary hosts). During parasitoid oviposition, PDVs enter infected secondary hosts, triggering expression of virulence genes, which manipulate multiple biological processes of hosts to fulfill all the requirements of parasitoid offspring. Bracovirus circles are integrated into host genomic DNA after natural parasitization. For ichnoviruses, the ability of the viral molecules to integrate in lepidopteran cell lines has also been shown, but there was no knowledge on what succeeds *in vivo*. We found that ichnovirus circles were present in hosts with non-integrated and integrated forms, and ichnoviruses integrated DNA circles into the host genome by two distinct strategies, conservatively and randomly. We identified the breaking sites of ichnovirus circles and the integration sites in the host genome. Our work shows, for the first time, the integration of ichnovirus molecules in the genome of the lepidopteran host following parasitism, which will deepen our understanding of how PDV circles persist in hosts and integrate into host genomic DNA.

## Data Availability Statement

The datasets presented in this study can be found in online repositories. The names of the repository/repositories and accession number(s) can be found below: https://www.ncbi.nlm.nih.gov/, SRR11880655, https://www.ncbi.nlm.nih.gov/, SRR11880656, https://www.ncbi.nlm.nih.gov/, SRR11880657.

## Author Contributions

X-xC, J-hH, and MS designed the study. Z-hW, Y-nZ, and X-qY performed the experiments and analyzed the data. Z-hW and X-xC wrote the manuscript. All authors reviewed the manuscript, contributed to the article, and approved the submitted version.

## Conflict of Interest

The authors declare that the research was conducted in the absence of any commercial or financial relationships that could be construed as a potential conflict of interest.
