# Supplementary material for: Genome-Wide Profiling of Diadegma semiclausum Ichnovirus Integration in Parasitized Plutella xylostella Hemocytes Identifies Host Integration Motifs and Insertion Sites
Source: Front Microbiol. 2021 Jan 15;11:608346. doi: 10.3389/fmicb.2020.608346 (PMC7843510; doi:10.3389/fmicb.2020.608346)
Supplement: Supplementary file 2 [file Image_1.PDF]

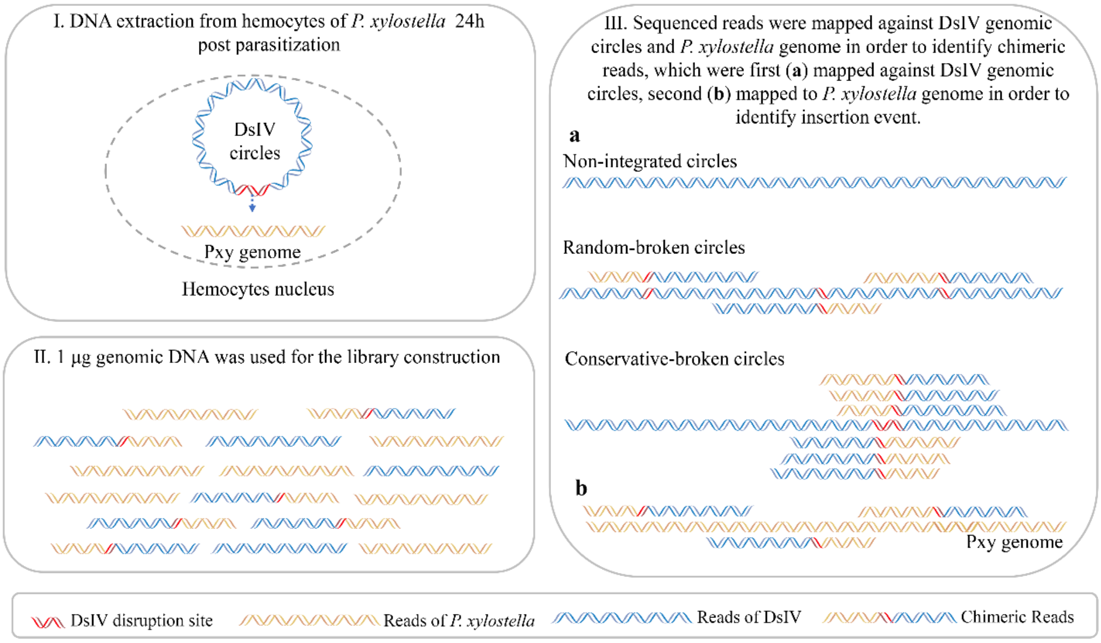

Fig. S1 Principle of the genome resequencing method used to detect integrations of DsIV circles in the *P. xylostella* genome. Step I: DNA was extracted from hemocytes of caterpillar hosts 24 h pp. The DNA used for sequencing consists of DsIV DNA, *P. xylostella* DNA, and *P. xylostella* DNA with integrated DsIV DNA. Step II: Chimeric reads containing both nucleotides of DsIV and *P. xylostella* sequences were sorted out based on BLASTN. Step III: Sequenced reads were mapped against DsIV genomic circles and *P. xylostella* genome in order to identify chimeric reads, which were first (**a**) mapped against DsIV genomic circles, second (**b**) mapped to *P. xylostella* genome in order to identify insertion event. DsIV circles were divided into 3 categories, non-integrated circles, random-broken circles, and conservative-broken circles.

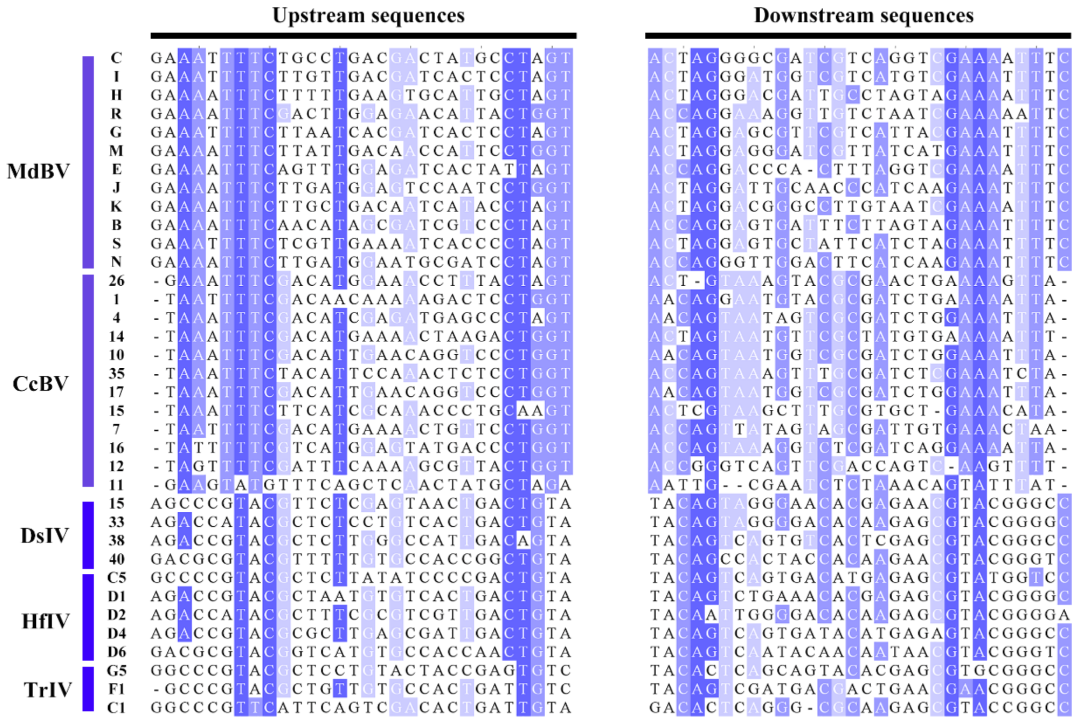

Fig. S2 Alignment of host integration motifs (HIMs) of BV circles and IV circles. Alignment of HIM sequences with similarity for each site colored in shades of blue for MdBV, CcBV, DsIV, HfIV, and TrIV.
