# Supplementary material for: Genome-Wide Profiling of Diadegma semiclausum Ichnovirus Integration in Parasitized Plutella xylostella Hemocytes Identifies Host Integration Motifs and Insertion Sites
Source: Front Microbiol. 2021 Jan 15;11:608346. doi: 10.3389/fmicb.2020.608346 (PMC7843510; doi:10.3389/fmicb.2020.608346)
Supplement: Supplementary file 3 [file Table_1.DOCX]

**Table S1 The basic features of sequence raw data of *P. xylostella* hemocytes at 24h pp by *D. semiclausum***

| **Samples** | **Ds-P-H-1** | **Ds-P-H-2** | **Ds-P-H-3** |
| --- | --- | --- | --- |
| Raw Reads Number | 637,738,012 | 668,183,680 | 534,371,170 |
| Raw Bases Number | 95,660,701,800 | 100,227,552,000 | 80,155,675,500 |
| Clean Reads Number | 410,675,546 | 591,336,836 | 403,959,804 |
| Clean Reads Rate (%) | 64.4 | 88.5 | 75.59 |
| Clean Bases Number | 61,601,331,900 | 88,700,525,400 | 60,593,970,600 |
| Raw Q30 Bases Rate (%) | 92.03 | 90.11 | 90.93 |
| Clean Q30 Bases Rate (%) | 92.42 | 90.39 | 91.29 |

Ds-P-H: Hemocytes of *P. xylostella* parasitized by *D. semiclausum.* Clean reads: valid reads filter from raw reads.

**Table S2 The percent of the integrated form of each DsIV circle**

| **DsIV ID** | **circle size (bp)** | **Ds-P-H-1** | **Ds-P-H-2** | **Ds-P-H-3** | **Total reads** | **The depth (×)** | **Chimeric reads** | **The percent of integrated form (%)** |
| --- | --- | --- | --- | --- | --- | --- | --- | --- |
| DsIV-01 | 5779 | 33697 | 46124 | 33916 | 113737 | 2952.16 | 0 | 0.00 |
| DsIV-02 | 4225 | 63063 | 86417 | 65129 | 214609 | 7619.25 | 8 | 0.05 |
| DsIV-03 | 3409 | 37080 | 49024 | 38839 | 124943 | 5497.64 | 3 | 0.03 |
| DsIV-04 | 3827 | 57469 | 76645 | 58756 | 192870 | 7559.58 | 12 | 0.08 |
| DsIV-05 | 3531 | 2456 | 3629 | 2735 | 8820 | 374.68 | 10 | 1.33 |
| DsIV-06 | 4794 | 10545 | 14151 | 10063 | 34759 | 1087.58 | 17 | 0.78 |
| DsIV-07 | 3968 | 2309 | 3055 | 2281 | 7645 | 289.00 | 36 | 6.23 |
| DsIV-08 | 4699 | 2924 | 3807 | 2930 | 9661 | 308.40 | 5 | 0.81 |
| DsIV-09 | 4838 | 6446 | 8367 | 6479 | 21292 | 660.15 | 17 | 1.29 |
| DsIV-10 | 4441 | 2417 | 3261 | 2619 | 8297 | 280.24 | 12 | 2.14 |
| DsIV-11 | 3559 | 2544 | 3733 | 2677 | 8954 | 377.38 | 9 | 1.19 |
| DsIV-12 | 2706 | 770 | 1018 | 719 | 2507 | 138.97 | 5 | 1.80 |
| DsIV-13 | 4021 | 372 | 587 | 421 | 1380 | 51.48 | 1 | 0.97 |
| DsIV-14 | 5315 | 5547 | 8036 | 5843 | 19426 | 548.24 | 7 | 0.64 |
| DsIV-15 | 4024 | 6075 | 7974 | 5624 | 19673 | 733.34 | 541 | 36.89 |
| DsIV-16 | 3418 | 256 | 340 | 281 | 877 | 38.49 | 3 | 3.90 |
| DsIV-17 | 5450 | 1510 | 2180 | 1598 | 5288 | 145.54 | 33 | 11.34 |
| DsIV-18 | 4031 | 7056 | 9554 | 6947 | 23557 | 876.59 | 14 | 0.80 |
| DsIV-19 | 4384 | 6959 | 9142 | 6951 | 23052 | 788.73 | 24 | 1.52 |
| DsIV-20 | 3373 | 2933 | 3931 | 3092 | 9956 | 442.75 | 0 | 0.00 |
| DsIV-21 | 4505 | 5264 | 6741 | 5293 | 17298 | 575.96 | 69 | 5.99 |
| DsIV-22 | 3545 | 2087 | 2863 | 2356 | 7306 | 309.14 | 6 | 0.97 |
| DsIV-23 | 4961 | 9544 | 13362 | 9619 | 32525 | 983.42 | 17 | 0.86 |
| DsIV-24 | 3917 | 3796 | 5049 | 4066 | 12911 | 494.42 | 28 | 2.83 |
| DsIV-25 | 5239 | 8726 | 11670 | 8973 | 29369 | 840.88 | 14 | 0.83 |
| DsIV-26 | 4394 | 7260 | 10144 | 7393 | 24797 | 846.51 | 25 | 1.48 |
| DsIV-27 | 2875 | 2387 | 3314 | 2498 | 8199 | 427.77 | 1 | 0.12 |
| DsIV-28 | 2900 | 3783 | 5526 | 3893 | 13202 | 682.86 | 21 | 1.54 |
| DsIV-29 | 5738 | 7147 | 10311 | 7727 | 25185 | 658.37 | 15 | 1.14 |
| DsIV-30 | 3018 | 2203 | 2793 | 2131 | 7127 | 354.22 | 18 | 2.54 |
| DsIV-31 | 3307 | 2071 | 2753 | 2115 | 6939 | 314.74 | 21 | 3.34 |
| DsIV-32 | 3609 | 5750 | 7910 | 6347 | 20007 | 831.55 | 1 | 0.06 |
| DsIV-33 | 5277 | 4807 | 6536 | 4991 | 16334 | 464.30 | 129 | 13.89 |
| DsIV-34 | 4065 | 3071 | 4281 | 3079 | 10431 | 384.91 | 9 | 1.17 |
| DsIV-35 | 3540 | 2981 | 3815 | 332 | 7128 | 302.03 | 31 | 5.13 |
| DsIV-36 | 2239 | 1228 | 1662 | 1339 | 4229 | 283.32 | 23 | 4.06 |
| DsIV-37 | 3821 | 5388 | 6659 | 5312 | 17359 | 681.46 | 0 | 0.00 |
| DsIV-38 | 4756 | 4426 | 5716 | 4744 | 14886 | 469.49 | 47 | 5.01 |
| DsIV-39 | 3640 | 2981 | 3983 | 3183 | 10147 | 418.15 | 13 | 1.55 |
| DsIV-40 | 5127 | 4894 | 6493 | 5263 | 16650 | 487.13 | 42 | 4.31 |
| DsIV-41 | 4203 | 5328 | 7024 | 5520 | 17872 | 637.83 | 35 | 2.74 |
| DsIV-42 | 7669 | 2089 | 2582 | 2114 | 6785 | 132.71 | 48 | 18.08 |
| DsIV-43 | 2800 | 3794 | 5336 | 3823 | 12953 | 693.91 | 3 | 0.22 |
| DsIV-44 | 3866 | 3353 | 4634 | 3630 | 11617 | 450.74 | 17 | 1.89 |
| DsIV-45 | 7529 | 8276 | 10896 | 8319 | 27491 | 547.70 | 13 | 1.19 |
| DsIV-46 | 5380 | 3512 | 4707 | 3768 | 11987 | 334.21 | 15 | 2.24 |
| DsIV-47 | 4597 | 4250 | 5786 | 4727 | 14763 | 481.72 | 17 | 1.76 |

Ds-P-H: Hemocytes of *P. xylostella* parasitized by *D. semiclausum*

**Table S3 The percent of different forms of 4 CBCs**

| **DsIV ID** | **Circular forms (%)** | **Conserved integrated form (%)** | **Randomly integrated form (%)** |
| --- | --- | --- | --- |
| DsIV-15 | 63.11 | 34.44 | 2.44 |
| DsIV-33 | 86.11 | 12.38 | 1.51 |
| DsIV-38 | 94.99 | 4.37 | 0.64 |
| DsIV-40 | 95.69 | 2.77 | 1.54 |

**Table S4 List of primers used in this study**

| **Primer name** | **Primer sequences (5’-3’)** | **Use** |
| --- | --- | --- |
| DsIV13-P-90 | GCAAGTCGCCATAATGAATGT | PCR-based assays |
| DsIV13-AP-1141 | CACATAGCACGTAGAGCTGACTG | PCR-based assays |
| DsIV13-P-1116 | CGTCAGTCAGCTCTACGTGCTAT | PCR-based assays |
| DsIV13-AP-2175 | GCTGTATCCATATGCAGGGTC | PCR-based assays |
| DsIV13-P-2120 | AGGCAGAGTGCAACAACAGG | PCR-based assays |
| DsIV13-AP-3105 | CCAGGAGAACTACGAGGGACT | PCR-based assays |
| DsIV13-P-2984 | GGTACGCAACAACTATTCCACG | PCR-based assays |
| DsIV13-AP-131 | GCAGCGTCATTCGTCATCG | PCR-based assays |
| DsIV15-p-187 | TCGTTGTCAAGAAATCCTCGG | PCR-based assays |
| DsIV15-ap-1125 | CCAGTAGTTGCGAATCCAGGT | PCR-based assays |
| DsIV15-p-1034 | ACAACCACGGCGTGAACTAG | PCR-based assays |
| DsIV15-ap-2244 | CTGCCGCCGTTTATCTTCTT | PCR-based assays |
| DsIV15-p-2021 | ACGGGCCGTAATTCCTAGAT | PCR-based assays |
| DsIV15-ap-3136 | CGGCGACTGTGCTATCTTTG | PCR-based assays |
| DsIV15-p-3032 | GAACTTTTTGTATGTTGCGGAC | PCR-based assays |
| DsIV15-ap-246 | GGTCTGGACCACTGTCAAATC | PCR-based assays |
| DsIV21-P-26 | AGAGCCACCGACTTTCTTATC | PCR-based assays |
| DsIV21-AP-1045 | CACTGCTATGCTCGTAACTTCAG | PCR-based assays |
| DsIV21-P-1002 | TTGGCATGTTGGAAAGTGAAG | PCR-based assays |
| DsIV21-AP-2081 | GTGGAACGAACGACGAATG | PCR-based assays |
| DsIV21-P-1994 | CGTTGGGTTTGAGTATGTCTGC | PCR-based assays |
| DsIV21-AP-3092 | TGTTCCAGCTGGCTTATGTTG | PCR-based assays |
| DsIV21-P-2980 | TCTCTCCATTCGGCAGATTATT | PCR-based assays |
| DsIV21-AP-4092 | CTGAAGAACAAGTCCAGGCTATAC | PCR-based assays |
| DsIV21-P-3872 | CATGGTTATTTCGTCAGGTGTG | PCR-based assays |
| DsIV21-AP-414 | CCGCGTTGATTGTGAGACTT | PCR-based assays |
| DsIV40-P-156 | GCCATTGTTGCCGTAAGTG | PCR-based assays |
| DsIV40-AP-1126 | CGAACAGCGTGCATACATCAT | PCR-based assays |
| DsIV40-P-1020 | CTGCTATTTGTGCACTCATCGT | PCR-based assays |
| DsIV40-AP-2111 | TCTTAGGCTGAAACGTGGGT | PCR-based assays |
| DsIV40-P-2092 | ACCCACGTTTCAGCCTAAGAT | PCR-based assays |
| DsIV40-AP-3169 | TGGAAAGACCGAATATTGCTC | PCR-based assays |
| DsIV40-P-3033 | AACACGGCCATTTTCAACACT | PCR-based assays |
| DsIV40-AP-4065 | CATCGGTTGCCAATCTTACG | PCR-based assays |
| DsIV40-P-4002 | CACGAGCATAGAGCCAGAGTC | PCR-based assays |
| DsIV40-AP-164 | AACAATGGCGACTCTAAGCG | PCR-based assays |
| D2-D3A-P | GCGAACAAGTACCGTGAGGG | 28S rRNA |
| D2-D3A-AP | TAGTTCACCATCTTTCGGGTC | 28S rRNA |

**Table S5 List of the accession numbers of other IV circles**

| **BVs** | **Accession number** | **circle ID** | **BVs** | **Accession number** | **circle ID** | **BVs** | **Accession number** | **circle ID** | **BVs** | **Accession number** | **circle ID** | **BVs** | **Accession number** | **circle ID** |
| --- | --- | --- | --- | --- | --- | --- | --- | --- | --- | --- | --- | --- | --- | --- |
| HfIV | AB291197.1 | D2 | TrIV | AB291214.1 | c289 | GfIV | AB289920.1 | B8 | AsIV | KC752384.1 | 179 | AsIV | KC752294.1 | 88 |
| HfIV | AY597814.1 | B8 | TrIV | AB291213.1 | c166 | GfIV | AB289919.1 | B7 | AsIV | KC752383.1 | 170 | AsIV | KC752293.1 | 87 |
| HfIV | AY577429.1 | C10 | TrIV | AB291215.1 | c111 | GfIV | AB289918.1 | B6 | AsIV | KC752382.1 | 177 | AsIV | KC752292.1 | 86 |
| HfIV | AY577428.1 | B17 | GfIV | AB290007.1 | E1 | GfIV | AB289917.1 | B5 | AsIV | KC752381.1 | 176 | AsIV | KC752291.1 | 85 |
| HfIV | AY570799.1 | C2 | GfIV | AB290006.1 | D7 | GfIV | AB289916.1 | B4 | AsIV | KC752380.1 | 175 | AsIV | KC752290.1 | 84 |
| HfIV | AY570798.1 | B11 | GfIV | AB290005.1 | D6 | GfIV | AB289915.1 | B3 | AsIV | KC752379.1 | 174 | AsIV | KC752289.1 | 83 |
| HfIV | AY563519.1 | D5 | GfIV | AB290004.1 | D5 | GfIV | AB289914.1 | B2 | AsIV | KC752378.1 | 173 | AsIV | KC752288.1 | 82 |
| HfIV | AY563518.1 | B7 | GfIV | AB290003.1 | D4 | GfIV | AB289913.1 | B1 | AsIV | KC752377.1 | 172 | AsIV | KC752287.1 | 81 |
| HfIV | AY556384.1 | C12 | GfIV | AB290002.1 | D3 | GfIV | AB289912.1 | A10 | AsIV | KC752376.1 | 171 | AsIV | KC752286.1 | 80 |
| HfIV | AY556383.1 | A1 | GfIV | AB290001.1 | D2 | GfIV | AB289911.1 | A9 | AsIV | KC752375.1 | 169 | AsIV | KC752285.1 | 79 |
| HfIV | AY547319.1 | C16 | GfIV | AB290000.1 | D1 | GfIV | AB289910.1 | A8 | AsIV | KC752374.1 | 168 | AsIV | KC752284.1 | 78 |
| HfIV | AB291209.1 | G1 | GfIV | AB289999.1 | C22 | GfIV | AB289909.1 | A7 | AsIV | KC752373.1 | 167 | AsIV | KC752283.1 | 77 |
| HfIV | AB291208.1 | E2 | GfIV | AB289998.1 | C21 | GfIV | AB289908.1 | A6 | AsIV | KC752372.1 | 166 | AsIV | KC752282.1 | 76 |
| HfIV | AB291207.1 | E1 | GfIV | AB289997.1 | C20 | GfIV | AB289907.1 | A5 | AsIV | KC752371.1 | 165 | AsIV | KC752281.1 | 75 |
| HfIV | AB291206.1 | D12 | GfIV | AB289996.1 | C19 | GfIV | AB289906.1 | A4 | AsIV | KC752370.1 | 164 | AsIV | KC752280.1 | 74 |
| HfIV | AB291205.1 | D11 | GfIV | AB289995.1 | C18 | GfIV | AB289905.1 | A3 | AsIV | KC752369.1 | 163 | AsIV | KC752279.1 | 73 |
| HfIV | AB291204.1 | D10 | GfIV | AB289994.1 | C17 | GfIV | AB289904.1 | A2 | AsIV | KC752368.1 | 162 | AsIV | KC752278.1 | 72 |
| HfIV | AB291203.1 | D9 | GfIV | AB289993.1 | C16 | GfIV | AB289903.1 | A1 | AsIV | KC752367.1 | 161 | AsIV | KC752277.1 | 71 |
| HfIV | AB291202.1 | D8 | GfIV | AB289992.1 | C15 | GfIV | AB295392.1 | B55 | AsIV | KC752366.1 | 160 | AsIV | KC752276.1 | 70 |
| HfIV | AB291201.1 | D7 | GfIV | AB289991.1 | C14 | CsIV | NC_008007.1 | W | AsIV | KC752365.1 | 159 | AsIV | KC752275.1 | 69 |
| HfIV | AB291200.1 | D6 | GfIV | AB289990.1 | C13 | CsIV | U41655.2 | V | AsIV | KC752364.1 | 158 | AsIV | KC752274.1 | 68 |
| HfIV | AB291199.1 | D4 | GfIV | AB289989.1 | C12 | CsIV | AF361487.1 | Q | AsIV | KC752363.1 | 157 | AsIV | KC752273.1 | 67 |
| HfIV | AB291198.1 | D3 | GfIV | AB289988.1 | C11 | CsIV | AY029394.2 | N | AsIV | KC752362.1 | 156 | AsIV | KC752272.1 | 66 |
| HfIV | AB291196.1 | D1 | GfIV | AB289987.1 | C10 | CsIV | AF361488.1 | F | AsIV | KC752361.1 | 155 | AsIV | KC752271.1 | 65 |
| HfIV | AB291195.1 | C20 | GfIV | AB289986.1 | C9 | CsIV | AY029400.1 | Z | AsIV | KC752360.1 | 154 | AsIV | KC752270.1 | 64 |
| HfIV | AB291194.1 | C19 | GfIV | AB289985.1 | C8 | CsIV | AY029398.1 | U | AsIV | KC752359.1 | 153 | AsIV | KC752269.1 | 63 |
| HfIV | AB291193.1 | C18 | GfIV | AB289984.1 | C7 | CsIV | AY029397.1 | T | AsIV | KC752358.1 | 152 | AsIV | KC752268.1 | 62 |
| HfIV | AB291192.1 | C17 | GfIV | AB289983.1 | C6 | CsIV | AY029396.1 | P | AsIV | KC752357.1 | 151 | AsIV | KC752267.1 | 61 |
| HfIV | AB291191.1 | C15 | GfIV | AB289982.1 | C5 | CsIV | AY029395.1 | O1 | AsIV | KC752356.1 | 150 | AsIV | KC752266.1 | 60 |
| HfIV | AB291190.1 | C14 | GfIV | AB289981.1 | C4 | CsIV | AF411011.1 | A | AsIV | KC752355.1 | 149 | AsIV | KC752265.1 | 59 |
| HfIV | AB291189.1 | C13 | GfIV | AB289980.1 | C3 | CsIV | AF362517.1 | M | AsIV | KC752354.1 | 148 | AsIV | KC752264.1 | 58 |
| HfIV | AB291188.1 | C11 | GfIV | AB289979.1 | C2 | CsIV | AF362516.1 | I2 | AsIV | KC752353.1 | 147 | AsIV | KC752263.1 | 57 |
| HfIV | AB291187.1 | C9 | GfIV | AB289978.1 | C1 | CsIV | AF362515.1 | L | AsIV | KC752352.1 | 146 | AsIV | KC752262.1 | 56 |
| HfIV | AB291186.1 | C8 | GfIV | AB289977.1 | B65 | CsIV | AF362514.1 | J | AsIV | KC752351.1 | 145 | AsIV | KC752261.1 | 55 |
| HfIV | AB291185.1 | C7 | GfIV | AB289976.1 | B64 | CsIV | AF362513.1 | I | AsIV | KC752350.1 | 144 | AsIV | KC752260.1 | 54 |
| HfIV | AB291184.1 | C6 | GfIV | AB289975.1 | B63 | CsIV | AF362512.1 | H | AsIV | KC752349.1 | 143 | AsIV | KC752259.1 | 53 |
| HfIV | AB291183.1 | C5 | GfIV | AB289974.1 | B62 | CsIV | AF362511.1 | G2 | AsIV | KC752348.1 | 142 | AsIV | KC752258.1 | 52 |
| HfIV | AB291182.1 | C4 | GfIV | AB289973.1 | B61 | CsIV | AF362510.1 | G | AsIV | KC752347.1 | 141 | AsIV | KC752257.1 | 51 |
| HfIV | AB291181.1 | C3 | GfIV | AB289972.1 | B60 | CsIV | AF362509.1 | E | AsIV | KC752346.1 | 140 | AsIV | KC752256.1 | 50 |
| HfIV | AB291180.1 | C1 | GfIV | AB289971.1 | B59 | CsIV | AF362508.1 | B | AsIV | KC752345.1 | 139 | AsIV | KC752255.1 | 49 |
| HfIV | AB291179.1 | B18 | GfIV | AB289970.1 | B58 | CsIV | AF362507.1 | C | AsIV | KC752344.1 | 138 | AsIV | KC752254.1 | 48 |
| HfIV | AB291178.1 | B16 | GfIV | AB289969.1 | B57 | CsIV | AF361869.1 | D | AsIV | KC752343.1 | 137 | AsIV | KC752253.1 | 47 |
| HfIV | AB291177.1 | B15 | GfIV | AB289968.1 | B56 | AsIV | KC752432.1 | 227 | AsIV | KC752342.1 | 136 | AsIV | KC752252.1 | 46 |
| HfIV | AB291176.1 | B14 | GfIV | AB289967.1 | B55 | AsIV | KC752431.1 | 226 | AsIV | KC752341.1 | 135 | AsIV | KC752251.1 | 45 |
| HfIV | AB291175.1 | B13 | GfIV | AB289966.1 | B54 | AsIV | KC752430.1 | 225 | AsIV | KC752340.1 | 134 | AsIV | KC752250.1 | 44 |
| HfIV | AB291174.1 | B12 | GfIV | AB289965.1 | B53 | AsIV | KC752429.1 | 224 | AsIV | KC752339.1 | 133 | AsIV | KC752249.1 | 43 |
| HfIV | AB291173.1 | B10 | GfIV | AB289964.1 | B52 | AsIV | KC752428.1 | 223 | AsIV | KC752338.1 | 132 | AsIV | KC752248.1 | 42 |
| HfIV | AB291172.1 | B9 | GfIV | AB289963.1 | B51 | AsIV | KC752427.1 | 222 | AsIV | KC752337.1 | 131 | AsIV | KC752247.1 | 41 |
| HfIV | AB291171.1 | B6 | GfIV | AB289962.1 | B50 | AsIV | KC752426.1 | 221 | AsIV | KC752336.1 | 130 | AsIV | KC752246.1 | 40 |
| HfIV | AB291170.1 | B5 | GfIV | AB289961.1 | B49 | AsIV | KC752425.1 | 220 | AsIV | KC752335.1 | 129 | AsIV | KC752245.1 | 39 |
| HfIV | AB291169.1 | B4 | GfIV | AB289960.1 | B48 | AsIV | KC752424.1 | 219 | AsIV | KC752334.1 | 128 | AsIV | KC752244.1 | 38 |
| HfIV | AB291168.1 | B3 | GfIV | AB289959.1 | B47 | AsIV | KC752423.1 | 218 | AsIV | KC752333.1 | 127 | AsIV | KC752243.1 | 37 |
| HfIV | AB291167.1 | B2 | GfIV | AB289958.1 | B46 | AsIV | KC752422.1 | 217 | AsIV | KC752332.1 | 126 | AsIV | KC752242.1 | 36 |
| HfIV | AB291166.1 | A3 | GfIV | AB289957.1 | B45 | AsIV | KC752421.1 | 216 | AsIV | KC752331.1 | 125 | AsIV | KC752241.1 | 35 |
| HfIV | AB291165.1 | A2 | GfIV | AB289956.1 | B44 | AsIV | KC752420.1 | 215 | AsIV | KC752330.1 | 124 | AsIV | KC752240.1 | 34 |
| HfIV | AY935249.1 | B1 | GfIV | AB289955.1 | B43 | AsIV | KC752419.1 | 214 | AsIV | KC752329.1 | 123 | AsIV | KC752239.1 | 33 |
| TrIV | KC176799.1 | G3-2 | GfIV | AB289954.1 | B42 | AsIV | KC752418.1 | 213 | AsIV | KC752328.1 | 122 | AsIV | KC752238.1 | 32 |
| TrIV | KC176798.1 | G2-2 | GfIV | AB289953.1 | B41 | AsIV | KC752417.1 | 212 | AsIV | KC752327.1 | 121 | AsIV | KC752237.1 | 31 |
| TrIV | AB291149.2 | D1 | GfIV | AB289952.1 | B40 | AsIV | KC752416.1 | 211 | AsIV | KC752326.1 | 120 | AsIV | KC752236.1 | 30 |
| TrIV | AB291161.2 | G3-1 | GfIV | AB289951.1 | B39 | AsIV | KC752415.1 | 210 | AsIV | KC752325.1 | 119 | AsIV | KC752235.1 | 29 |
| TrIV | AB291160.2 | G2-1 | GfIV | AB289950.1 | B38 | AsIV | KC752414.1 | 209 | AsIV | KC752324.1 | 118 | AsIV | KC752234.1 | 28 |
| TrIV | AB291164.2 | B3 | GfIV | AB289949.1 | B37 | AsIV | KC752413.1 | 208 | AsIV | KC752323.1 | 117 | AsIV | KC752233.1 | 27 |
| TrIV | AB291163.1 | G5 | GfIV | AB289948.1 | B36 | AsIV | KC752412.1 | 207 | AsIV | KC752322.1 | 116 | AsIV | KC752232.1 | 26 |
| TrIV | AB291162.1 | G4 | GfIV | AB289947.1 | B35 | AsIV | KC752411.1 | 206 | AsIV | KC752321.1 | 115 | AsIV | KC752231.1 | 25 |
| TrIV | AB291159.1 | G1 | GfIV | AB289946.1 | B34 | AsIV | KC752410.1 | 205 | AsIV | KC752320.1 | 114 | AsIV | KC752230.1 | 24 |
| TrIV | AB291158.1 | F3 | GfIV | AB289945.1 | B33 | AsIV | KC752409.1 | 204 | AsIV | KC752319.1 | 113 | AsIV | KC752229.1 | 23 |
| TrIV | AB291157.1 | F2 | GfIV | AB289944.1 | B32 | AsIV | KC752408.1 | 203 | AsIV | KC752318.1 | 112 | AsIV | KC752228.1 | 22 |
| TrIV | AB291156.1 | E1 | GfIV | AB289943.1 | B31 | AsIV | KC752407.1 | 202 | AsIV | KC752317.1 | 111 | AsIV | KC752227.1 | 21 |
| TrIV | AB291155.1 | D7 | GfIV | AB289942.1 | B30 | AsIV | KC752406.1 | 201 | AsIV | KC752316.1 | 110 | AsIV | KC752226.1 | 20 |
| TrIV | AB291154.1 | D6 | GfIV | AB289941.1 | B29 | AsIV | KC752405.1 | 200 | AsIV | KC752315.1 | 109 | AsIV | KC752225.1 | 19 |
| TrIV | AB291153.1 | D5 | GfIV | AB289940.1 | B28 | AsIV | KC752404.1 | 199 | AsIV | KC752314.1 | 108 | AsIV | KC752224.1 | 18 |
| TrIV | AB291152.1 | D4 | GfIV | AB289939.1 | B27 | AsIV | KC752403.1 | 198 | AsIV | KC752313.1 | 107 | AsIV | KC752223.1 | 17 |
| TrIV | AB291151.1 | D3 | GfIV | AB289938.1 | B26 | AsIV | KC752402.1 | 197 | AsIV | KC752312.1 | 106 | AsIV | KC752222.1 | 16 |
| TrIV | AB291150.1 | D2 | GfIV | AB289937.1 | B25 | AsIV | KC752401.1 | 196 | AsIV | KC752311.1 | 105 | AsIV | KC752221.1 | 15 |
| TrIV | AB291148.1 | C7 | GfIV | AB289936.1 | B24 | AsIV | KC752400.1 | 195 | AsIV | KC752310.1 | 104 | AsIV | KC752220.1 | 14 |
| TrIV | AB291147.1 | C7 | GfIV | AB289935.1 | B23 | AsIV | KC752399.1 | 194 | AsIV | KC752309.1 | 103 | AsIV | KC752219.1 | 13 |
| TrIV | AB291146.1 | C6 | GfIV | AB289934.1 | B22 | AsIV | KC752398.1 | 193 | AsIV | KC752308.1 | 102 | AsIV | KC752218.1 | 12 |
| TrIV | AB291145.1 | C5 | GfIV | AB289933.1 | B21 | AsIV | KC752397.1 | 192 | AsIV | KC752307.1 | 101 | AsIV | KC752217.1 | 11 |
| TrIV | AB291144.1 | C4 | GfIV | AB289932.1 | B20 | AsIV | KC752396.1 | 191 | AsIV | KC752306.1 | 100 | AsIV | KC752216.1 | 10 |
| TrIV | AB291143.1 | C3 | GfIV | AB289931.1 | B19 | AsIV | KC752395.1 | 190 | AsIV | KC752305.1 | 99 | AsIV | KC752215.1 | 9 |
| TrIV | AB291142.1 | C2 | GfIV | AB289930.1 | B18 | AsIV | KC752394.1 | 189 | AsIV | KC752304.1 | 98 | AsIV | KC752214.1 | 8 |
| TrIV | AB291141.1 | B2 | GfIV | AB289929.1 | B17 | AsIV | KC752393.1 | 188 | AsIV | KC752303.1 | 97 | AsIV | KC752213.1 | 7 |
| TrIV | AB291140.1 | B1 | GfIV | AB289928.1 | B16 | AsIV | KC752392.1 | 187 | AsIV | KC752302.1 | 96 | AsIV | KC752212.1 | 6 |
| TrIV | AB291139.1 | A2 | GfIV | AB289927.1 | B15 | AsIV | KC752391.1 | 186 | AsIV | KC752301.1 | 95 | AsIV | KC752211.1 | 5 |
| TrIV | AB291138.1 | A1 | GfIV | AB289926.1 | B14 | AsIV | KC752390.1 | 185 | AsIV | KC752300.1 | 94 | AsIV | KC752210.1 | 4 |
| TrIV | AF529168.2 | B4 | GfIV | AB289925.1 | B13 | AsIV | KC752389.1 | 184 | AsIV | KC752299.1 | 93 | AsIV | KC752209.1 | 3 |
| TrIV | AY940454.1 | C1 | GfIV | AB289924.1 | B12 | AsIV | KC752388.1 | 183 | AsIV | KC752298.1 | 92 | AsIV | KC752208.1 | 2 |
| TrIV | AF421353.1 | F1 | GfIV | AB289923.1 | B11 | AsIV | KC752387.1 | 182 | AsIV | KC752297.1 | 91 | AsIV | KC752207.1 | 1 |
| TrIV | AF052837.1 | 61 | GfIV | AB289922.1 | B10 | AsIV | KC752386.1 | 181 | AsIV | KC752296.1 | 90 |  |  |  |
| TrIV | AF052836.1 | 57 | GfIV | AB289921.1 | B9 | AsIV | KC752385.1 | 180 | AsIV | KC752295.1 | 89 |  |  |  |
